# Supplementary material for: Considerations about the Continuous Assay Methods, Spectrophotometric and Spectrofluorometric, of the Monophenolase Activity of Tyrosinase
Source: Biomolecules. 2021 Aug 25;11(9):1269. doi: 10.3390/biom11091269 (PMC8465126; doi:10.3390/biom11091269)
Supplement: Supplementary file 1 [file biomolecules-11-01269-s001.zip › biomolecules-1358976-final-suppl.pdf]

Considerations about the continuous assay methods, spectrophotometric and spectrofluorometric, of the monophenolase activity of tyrosinase.

Pablo García-Molina[1], José Luis Muñoz-Muñoz[2*], Joaquin A. Ortuño[3], José Neptuno Rodríguez-López[1], Pedro Antonio García-Ruiz[4], Francisco García-Cánovas[1] and Francisco García-Molina[5*]

[1]GENZ-Group of Research on Enzymology, Department of Biochemistry and Molecular Biology-A, Regional Campus of International Excellence "Campus Mare Nostrum", University of Murcia, Espinardo, Murcia, Spain.

[2]Microbiol Enzymology Lab, Department of Applied Sciences, Ellison Building A, University of Northumbria, Newcastle Upon Tyne, UK.

[3]Department of Analytical Chemistry, Faculty of Chemistry, University of Murcia, Murcia E-30100, Spain.

[4]Group of Chemistry of Carbohydrates, Industrial Polymers and Additives, Department of Organic Chemistry, Regional Campus of International Excellence "Campus Mare Nostrum", University of Murcia, E-30100, Espinardo, Murcia, Spain.

[5]Department of Anatomía Patológica. Hospital General Universitario Reina Sofía, Av. Intendente Jorge Palacios, 1, 30003 Murcia, Spain.

[*]Corresponding Authors: pacogm@um.es, jose.munoz@northumbria.ac.uk

SUPPLEMENTARY MATERIAL

Table of contents

Content of the materials.	Page
Scheme S 1. Diphenolase pathway. Structural mechanism.	S-4
Scheme S 2. Monophenolase pathway. Structural mechanism.	S-6
Kinetic analysis.	S-8
Steady-State Rate Equation for Diphenolase Activity.	S-8
Steady-State Rate Equation for Monophenolase Activity.	S-9
Transition phase.	S-10
Enzymatic reaction where the reaction product is attacked by a nucleophilic reagent: MBTH.	S-10
Scheme S 3. Diphenolase activity of tyrosinase in which the product, *o*-quinone, is attacked by a nucleophilic reagent such as MBTH.	S-11
Tyrosinase: Relationships between ligand rates.	S-12
Dipohenolase Activity.	S-12
a) *o*-Quinone, product of the enzymatic reaction, is stable. Scheme 2.	S-12
b) *o*-Quinone evolved to other product. Scheme 2.	S-12
c) *o*-Quinone evolved by attack of a nucleophilic reagent. Scheme S 3.	S-12
Monophenolase Activity. Differential Equations Corresponding to the Monophenolase Activity in Tyrosinase.	S-14
a) *o*-Quinone evolves to a product, which can be an aminochrome. Scheme 1.	S-14

b) *o*-Quinone evolves to a product, such as an oxidized adduct, after the attack of a nucleophile like MBTH.	S-14
Figures for the monophenolase activity.	S-16
Figure S1. Simulated curves of the action of tyrosinase on L-tyrosine, *o*-diphenol is not added to the medium.	S-17
Figure S2. Simulated curves of the action of tyrosinase on L-tyrosine, the *o*-diphenol necessary to reach the steady-state is added to the medium.	S-18
Figure S3. Simulated curves of the action of tyrosinase on L-tyrosine, a concentration of *o*-diphenol is added to the medium lower than that necessary to reach steady-state.	S-19
Figure S4. Simulated curves of the action of tyrosinase on L-tyrosine, a concentration of *o*-diphenol is added to the medium greater than that necessary to reach the steady-state.	S-21
Table S1. Kinetic characteristics of the action of tyrosinase on different monophenols and spectroscopic properties of the oxidized adducts formed with MBTH.	S-22
References.	S-23

Scheme S I

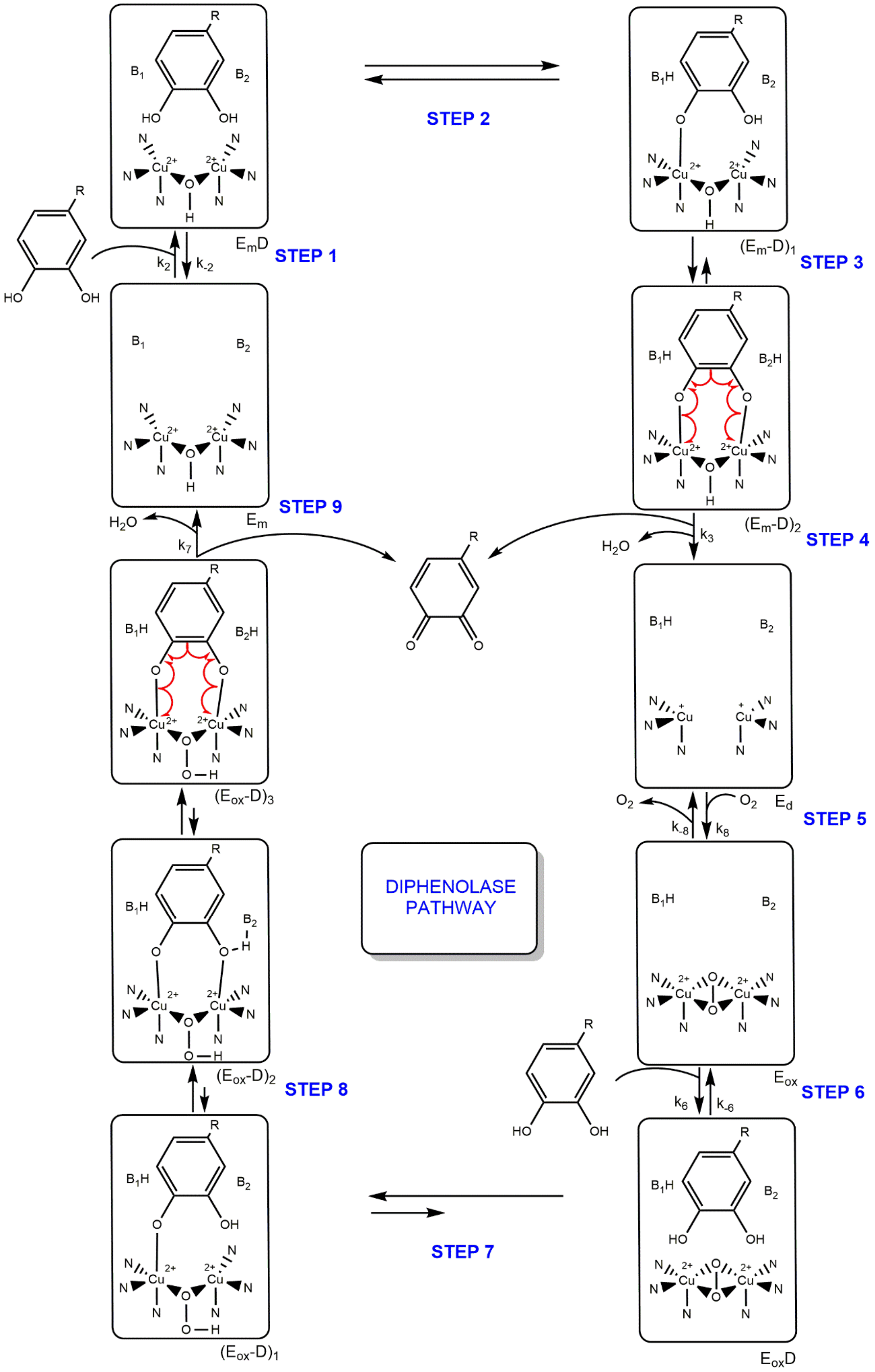

STEP 1
STEP 2
STEP 3
STEP 4
STEP 5
STEP 6
STEP 7
STEP 8
STEP 9
DIPHENOLASE PATHWAY
E_mD
$(E_m\text{-}D)_1$
$(E_m\text{-}D)_2$
E_m
E_d
E_{ox}
$E_{ox}D$
$(E_{ox}\text{-}D)_1$
$(E_{ox}\text{-}D)_2$
$(E_{ox}\text{-}D)_3$
k_2
k_{-2}
k_3
k_7
k_8
k_{-8}
k_6
k_{-6}
H_2O
O_2

Scheme S 1. Structural mechanism proposed to explain the diphenolase pathway of tyrosinase acting on *o*-diphenols. E_m, met-tyrosinase; E_d, deoxy-tyrosinase; E_{ox}, oxy-tyrosinase; $E_{ox}D$, oxy-tyrosinase/*o*-diphenol complex; $(E_{ox}\text{-}D)_1$, oxy-tyrosinase/*o*-diphenol complex axially bound to a Cu atom; $(E_{ox}\text{-}D)_2$, oxy-tyrosinase/*o*-diphenol complex axially bound to the two Cu atoms; E_mD, met-tyrosinase/*o*-diphenol complex; $(E_m\text{-}D)_1$, met-tyrosinase/*o*-diphenol complex axially bound to a Cu atom; $(E_m\text{-}D)_2$, met-tyrosinase/*o*-diphenol complex axially bound to the two Cu atoms [1].

Scheme S 2

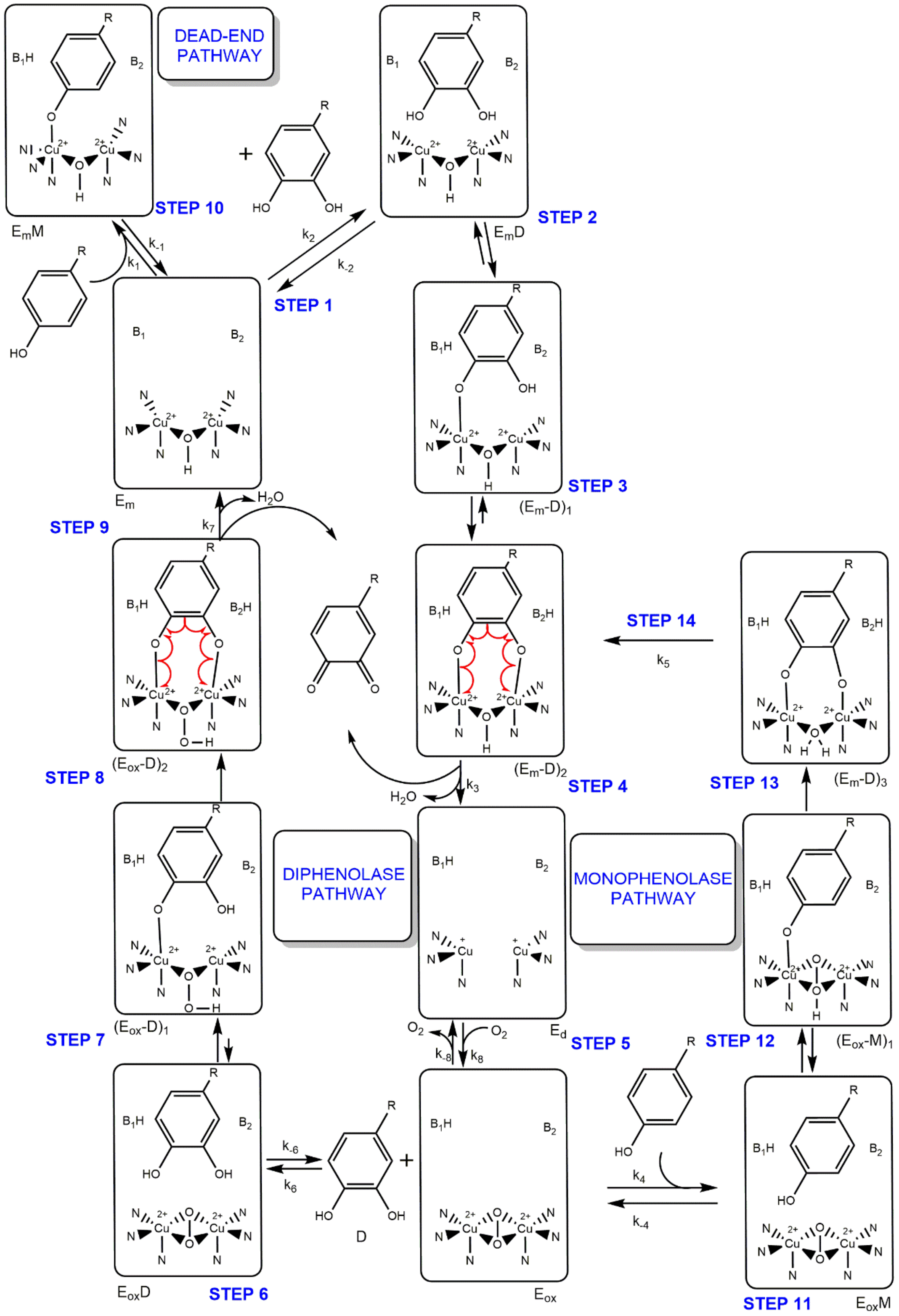

DEAD-END PATHWAY
DIPHENOLASE PATHWAY
MONOPHENOLASE PATHWAY
STEP 1
STEP 2
STEP 3
STEP 4
STEP 5
STEP 6
STEP 7
STEP 8
STEP 9
STEP 10
STEP 11
STEP 12
STEP 13
STEP 14
EmM
Em
EmD
(Em-D)1
(Em-D)2
(Em-D)3
Ed
Eox
EoxD
EoxM
(Eox-D)1
(Eox-D)2
(Eox-M)1
D
k1
k-1
k2
k-2
k3
k4
k-4
k5
k6
k-6
k7
k8
k-8
H2O
O2

Scheme S 2. Structural mechanism proposed to explain the diphenolase and monophenolase pathways of tyrosinase acting on monophenols and *o*-diphenols. E_m, met-tyrosinase; E_d, deoxy-tyrosinase; E_{ox}, oxy-tyrosinase; $E_{ox}D$, oxy-tyrosinase/*o*-diphenol complex; $(E_{ox}\text{-}D)_1$, oxy-tyrosinase/*o*-diphenol complex axially bound to a Cu atom; $(E_{ox}\text{-}D)_2$, oxy-tyrosinase/*o*-diphenol complex axially bound to the two Cu atoms; E_mD, met-tyrosinase/*o*-diphenol complex; $(E_m\text{-}D)_1$, met-tyrosinase/*o*-diphenol complex axially bound to a Cu atom; $(E_m\text{-}D)_2$, met-tyrosinase/*o*-diphenol complex axially bound to the two Cu atoms. $(E_m\text{-}D)_3$, met-tyrosinase/*o*-diphenol complex axial-equatorially bound to the two Cu atoms; E_mM, met-tyrosinase/monophenol complex; $E_{ox}M$, oxy-tyrosinase/monophenol complex; $(E_{ox}\text{-}M)_1$, oxy-tyrosinase/monophenol complex axially bound to a Cu atom [1].

1. Kinetic Analysis

2.1. Steady-State

2.1.1. Deduction of the Steady-State Rate Equation for Diphenolase Activity in Tyrosinase.

The structural mechanism for this activity is shown in Scheme S I and the kinetic scheme is indicated by Scheme II. The steady-state speed must comply with (the disappearance speed of *o*-diphenol (D) ($-V_{ss}^{D}$), must be equal to the rate of accumulation of dopachrome (DC) ($V_{ss}^{D,DC}$), and equal to the rate of oxygen disappearance ($-V_{ss}^{D,O_2}$):

$$-V_{ss}^{D,D} = V_{ss}^{D,DC} = -V_{ss}^{D,O_2} = \frac{\alpha_1[O_2]_0[D]_0[E]_0}{2(\beta_0+\beta_1[D]_0+\beta_2[O_2]_0+\beta_3[O_2]_0[D]_0)} \quad \text{(S1)}$$

Considering that the concentration of oxygen is saturating [2] $[O_2]_0 \rightarrow \infty$, the Equation S1 can be simplified as follows:

$$V_{ss}^{D,DC} = \frac{\alpha_1[D]_0[E]_0}{2(\beta_2+\beta_3[D]_0)} \quad \text{(S2)}$$

The expressions for α_1 and β_0 - β_3 are functions of the rate constants and can be obtained applying the programs described in [3].

Substituting these values in Equation (S2), and taking into account that,

$$k_3 \gg k_7, k_7 \gg k_{-6} \text{ y } k_3 \gg k_{-2}$$

we get:

$$V_{ss}^{D,DC} = \frac{V_{max}^{D,DC}[D]_0}{K_M^D+[D]_0} \quad \text{(S3)}$$

with:

$$V_{max}^{D,DC} = \frac{k_3k_7}{k_3+k_7}[E]_0 \cong k_7[E]_0 \quad \text{(S4)}$$

and:

$$K_m^D = \frac{k_2(k_2+k_6)}{k_2k_6} \quad \text{(S5)}$$

The binding of D to Em is easier than to Eox, so, $k_2 \gg k_6$ and K_m^D results in [4]:

$$K_m^D = \frac{k_7}{k_6} \qquad \text{(S6)}$$

2.1.2. Deduction of the Steady-State Rate Equation for Monophenolase Activity.

The structural mechanism of this activity is indicated in Scheme S2 and the kinetic scheme is indicated by Scheme 1. The steady-state rate is: (disappearance rate of M = formation rate of DC)

$$-V_{ss}^{M,M} = +V_{ss}^{M,DC} \qquad \text{(S7)}$$

With respect to oxygen, it is:

$$-V_{ss}^{M,O_2} = -1{,}5\ V_{ss}^{M,M} = +1{,}5\ V_{ss}^{M.DC} \qquad \text{(S8)}$$

Using the program described in [3], we can obtain:

$${}_{ss}^{M,DC} = \frac{[\alpha_1[O_2]_0[D]_0[M]_0+\alpha_2[O_2]_0[D]_0^2][E]_0}{2[\beta_1[D]_0+\beta_2[D]_0[M]_0+\beta_3[D]_0^2+\beta_4[O_2]_0[M]_0+\beta_5[O_2]_0[D]_0+\beta_6[O_2]_0[M]_0^2+\beta_7[O_2]_0[D]_0[M]_0+\beta_8[O_2]_0[D]_0^2]} \qquad \text{(S9)}$$

When $[O_2]_0 \rightarrow \infty$ [2].

$$V_{ss}^{M,DC} = \frac{[\alpha_1[D]_0[M]_0+\alpha_2[D]_0^2][E]_0}{2[\beta_4[M]_0+\beta_5[D]_0+\beta_6[M]_0^3+\beta_7[D]_0[M]_0+\beta_8[D]_0^2]} \qquad \text{(S10)}$$

and:

$$\frac{[D]_{ss}}{[M]_{ss}} = \frac{k_4k_5(k_{-6}+k_7)}{2k_7k_6(k_{-4}+k_5)} = R \qquad \text{(S11)}$$

Substituting Equation (S11) in Equation (S10), and considering the values of $\alpha_1 - \alpha_2$ and $\beta_1 - \beta_8$, obtained with the program described in [3],

We can obtain:

$$V_{ss}^{M,DC} = \frac{V_{max}^{M}[M]_0}{K_m^{M,DC}+[M]_0} = \frac{k_{cat}^{M,DC}[M]_0}{K_m^{M,DC}+[M]_0} \qquad \text{(S12)}$$

where

and if the limiting step is hydroxylating (governed by k_5), results:

$$k_5 \ll k_3, k_7 \quad \text{(S13)}$$

and can be obtained:

$$k_{cat}^{M,DC} = k_5 \quad \text{(S14)}$$

$$K_m^M = \frac{k_5}{k_4}\left(\frac{k_2+k_6}{k_2}\right) \quad \text{(S15)}$$

As

$$k_2 \gg k_6 \quad \text{(S16)}$$

results

$$K_m^M = \frac{k_5}{k_4} \quad \text{(S17)}$$

2.2. Transition phase

Next, we proposed the case where a transition phase and the subsequent arrival to the steady-state are shown:

Enzymatic reaction where the product is attacked by a nucleophilic reagent, the tyrosinase case.

Diphenolase activity in presence of a nucleophile. In the case of the diphenolase activity in tyrosinase, MBTH(N) has been used in many studies [5,6]. The scheme could be (Scheme S III):

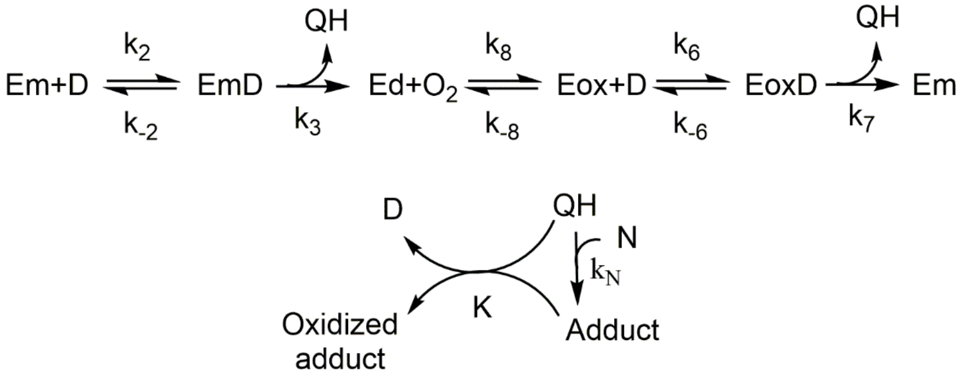

Scheme S III

The accumulation of the oxidized adduct over time is:

$$[A-ox]_t = \frac{V_{SS}}{2}\left(t + \frac{1}{k_N[N]}e^{-k_N[N]} - \frac{1}{k_N[N]}\right) \quad \text{(S18)}$$

and in the steady-state we obtain:

$$[A-ox]_{SS} = \frac{V_{SS}}{2}\left(t - \frac{1}{k_N[N]}\right) \quad \text{(S19)}$$

The kinetic analysis shows that the measured rate V_{ss} corresponds to the enzymatic reaction and since the system is in a steady-state, it is fulfilled:

$$-\frac{d[D]}{dt} = -\frac{d[O_2]}{dt} = +\frac{d[A-ox]}{dt} \quad \text{(S20)}$$

In this case, it is demonstrated by the analytical solutions obtained that it is not necessary to measure the disappearance of any substrate, if a coupled product or reagent can be easily measured.

2.3. Tyrosinase. Relationships between ligand rates.

2.3.1. Dipohenolase Activity.

The diphenolase activity in tyrosinase consists of the oxidation of two molecules from D to *o*-Q, this mechanism is linear, and its results are easy to be interpreted (Scheme II). According to the stability of the *o*-Q, we consider three cases:

a) Stable *o*-Q. This is the case for 4-*tert*-butylcatechol [7]. The mechanism

would be indicated in Scheme II, considering that *o*-Q, *o*-*tert*-butylquinone (*o*-TBQ) doesn't evolve. The mechanism following the stoichiometry 1 O_2 : 2 D : 2 Q, where D is TBC.

From these data it follows that:

$$-\frac{d[D]}{dt} = +\frac{d[Q]}{dt} = -2\frac{[O_2]}{dt} \qquad (S21)$$

b) Unstable *o*-Q. This is the case for the L-dopa oxidation [8].

o-Q evolves towards DC, note that D (L-dopa) is regenerated in the medium. Now, the mechanism follow the stoichiometry 1 O_2 : 1 D : 1 DC, where D is L-dopa.

From these results it follows that:

$$-\frac{d[D]}{dt} = +\frac{d[DC]}{dt} = -\frac{d[O_2]}{dt} \qquad (S22)$$

c) *o*-Q reacts with a nucelophilic reagent (N). The kinetic scheme is shown in Scheme S III.

In this case, *o*-Q reacts with a nucleophilic reagent (N) such as MBTH, an adduct is formed and that is oxidized to (A-ox) by another *o*-Q molecule [7,9].

Following the stoichiometry 1 O_2 : 1 D : 1 A-ox, from these results, it follows that:

$$-\frac{d[D]}{dt} = +\frac{d[A-ox]}{dt} = -\frac{d[O_2]}{dt} \qquad (S23)$$

In the three cases described, it is clearly shown that it is not necessary to measure the disappearance of substrate to obtain the rate of action of the enzyme in the steady-state. This rate of action of the enzyme can theoretically be achieved by measuring the D disappearance ($V_{SS}^{D,D}$), the O_2 disappearance (V_{SS}^{D,O_2}) or the DC formation ($V_{SS}^{D,DC}$) or the oxidized adduct $V_{SS}^{D,A-ox}$.

2.3.2. Monophenolase Activity. Differential Equations Corresponding to the Monophenolase Activity in Tyrosinase.

a) The mechanism of the monophenolase activity of tyrosinase (Scheme I),

considering that the *o*-quinone evolves to DC. The simulated mechanism correspond to the cases 1-4 [10]. The differential equations involved in the evolution of the different enzymatic species in these mechanisms are:

$$\left[\dot{\mathrm{Em}}\right] = \mathrm{k_{-2}[EmD] + k_7[EoxD] + k_{-1}[EmM] - k_2[D][Em] - k_1[M][Em]}$$

$$\left[\dot{\mathrm{Ed}}\right] = \mathrm{k_3[EmD] + k_{-8}[Eox] - k_8[O_2][Ed]}$$

$$\left[\dot{\mathrm{Eox}}\right] = \mathrm{k_8[O_2][Ed] + k_{-6}[EoxD] + k_{-4}[EoxM] - k_{-8}[EoxD] - k_6[D][Eox] - k_4[M][Eox]}$$

$$[\mathrm{E\dot{m}D}] = \mathrm{k_2[D][Em] + k_5[EoxM] - k_{-2}[EmD] - k_3[EmD]}$$

$$\left[\mathrm{Eo\dot{x}D}\right] = \mathrm{k_6[D][Eox] - k_7[EoxD] - k_{-6}[EoxD]}$$

$$\left[\mathrm{Eo\dot{x}M}\right] = \mathrm{k_4[M][Eox] - k_{-4}[EoxM] - k_5[EoxM]}$$

$[\dot{\text{EmM}}] = k_1[M][Em] - k_{-1}[EmM]$

$[\dot{Q}] = k_3[EmD] + k_7[EoxD] - 2k_{10}[Q]$

$[\dot{D}] = k_{-2}[EmD] + k_{-6}[EoxD] - k_2[D][Em] - k_6[D][Eox] + k_{10}[Q]$

$[\dot{\text{DC}}] = k_{10}[Q]$

$[\dot{M}] = k_{-1}[EmM] + k_{-4}[EoxM] - k_1[M][Em] - k_{-4}[M][Eox]$

$[\dot{O}_2] = k_{-8}[Eox] - k_8[O_2][Ed]$

b) Mechanism of the monophenolase activity of tyrosinase (Scheme I),

considering that the *o*-quinone evolves by the attack of a nucleophilic reagent (MBTH), originated from an adduct oxidized by another *o*-quinone molecule (case 5) [10]. The differential equations that show the evolution of the different enzymatic species are the same tan in case a) with some modifications:

$[\dot{Q}] = k_3[EmD] + k_7[EoxD] - 2k_{11}[N][Q]$

$[\dot{D}] = k_{-2}[EmD] + k_{-6}[EoxD] - k_2[D][Em] - k_6[D][Eox] + k_{11}[N][Q]$

$[Ad \dot{-} ox] = k_{11}[N][Q]$

$[\dot{N}] = -k_{11}[N][Q]$

Figures for Monophenolase Activity

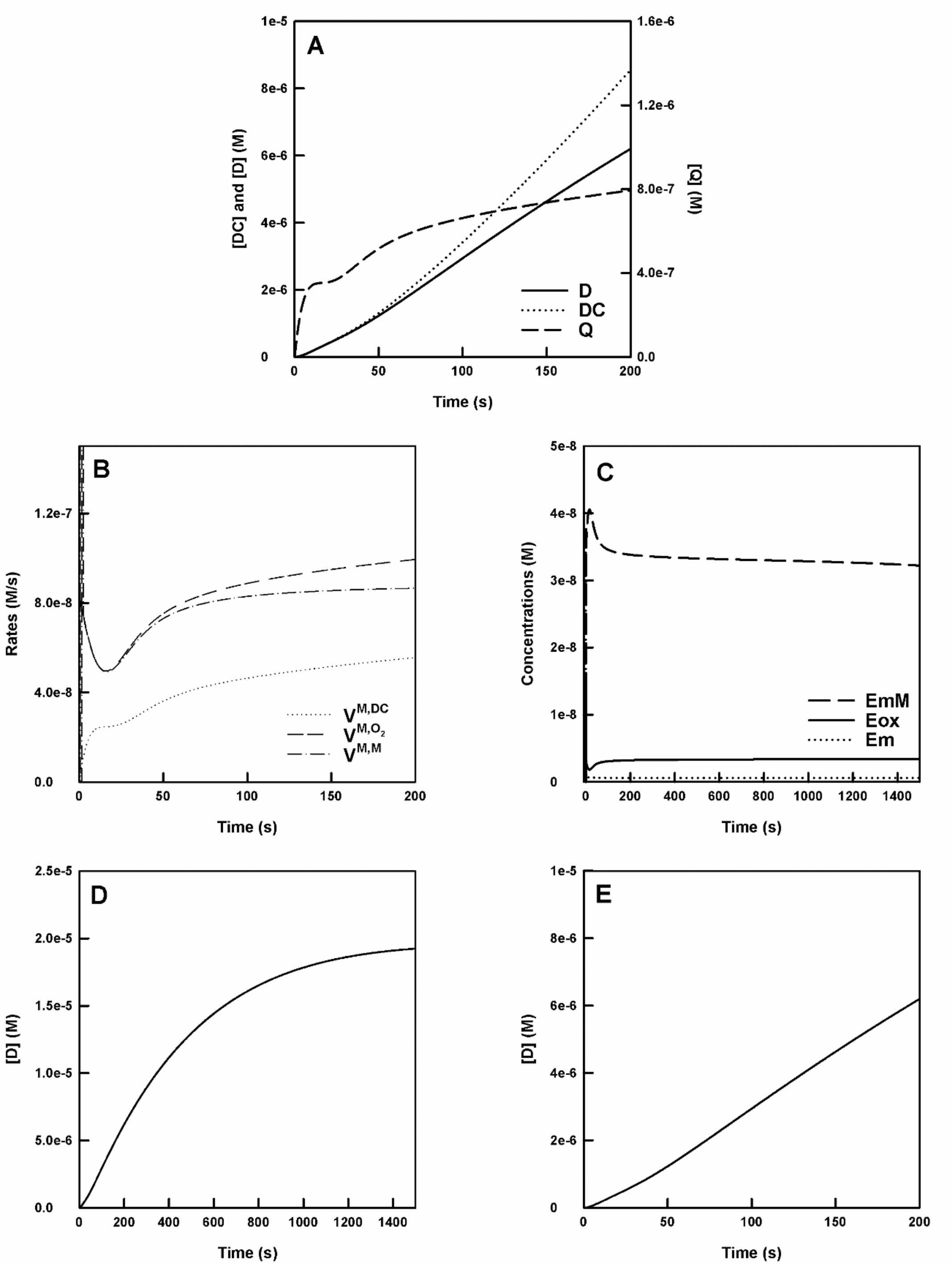

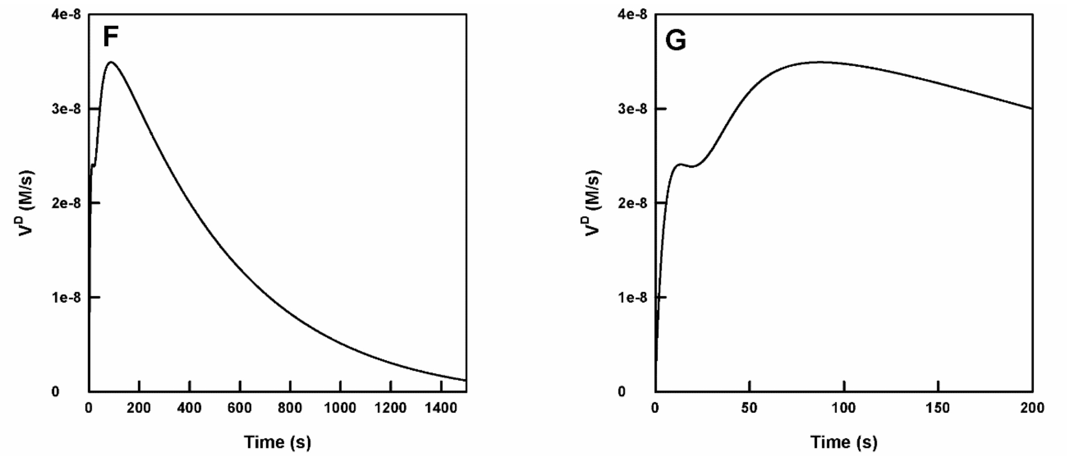

Figure S1. Simulated curves of the action of tyrosinase on L-tyrosine, *o*-diphenol is not added to the medium. A. Accumulation curves of DC, *o*-Q and D in short reaction times. B. Curves of the rates of consumption of O_2 (V^{M,O_2}), M ($V^{M,M}$) and of formation of DC ($V^{M,DC}$) at short reaction times. C. Evolution of the concentration of the different enzymatic forms: Eox, Em and EmM with the reaction time. D. Accumulation of D over time to a constant level upon arrival at steady-state. E. Accumulation of D at short reaction times. F. Representation of the rate of D accumulation over time. G. Variation of the rate of D accumulation with time, at the beginning of the reaction. The experimental conditions are the same than in Figure 1.

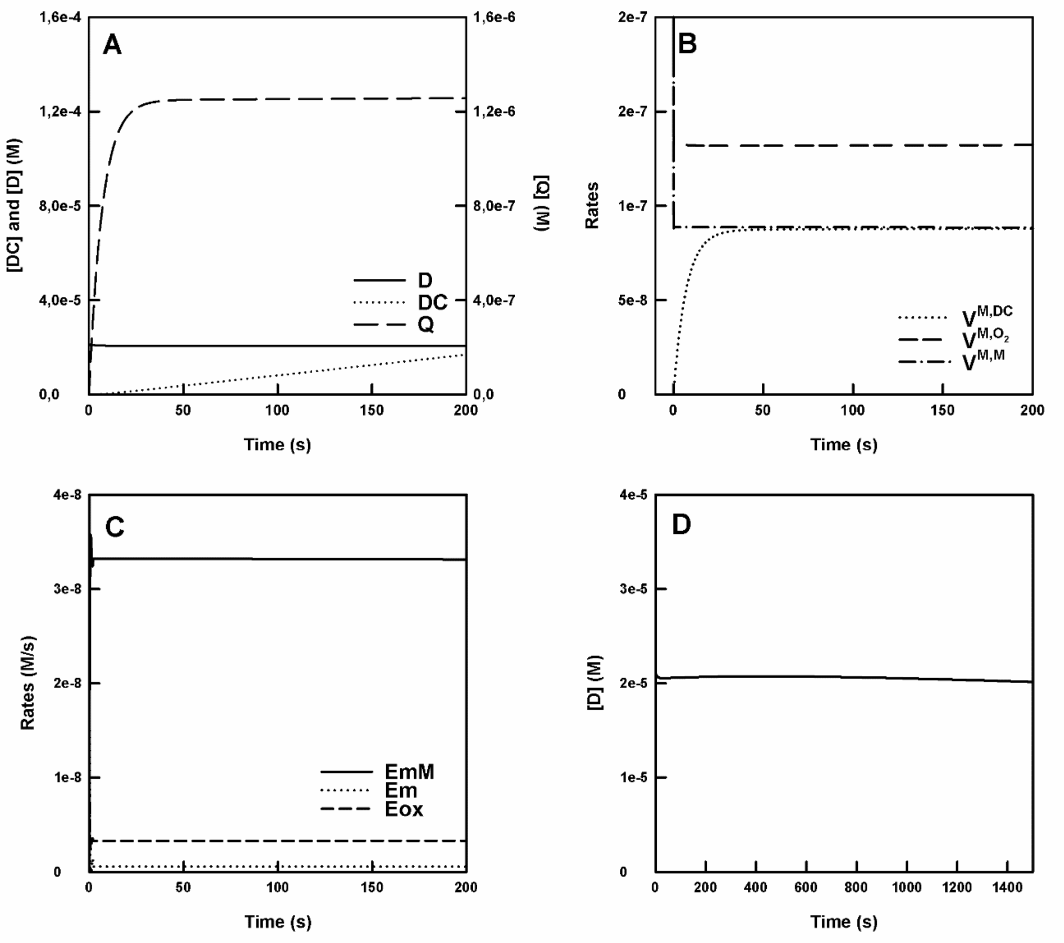

Figure S2. Simulated curves of the action of tyrosinase on L-tyrosine, the *o*-diphenol necessary to reach the steady-state is added to the medium. A. Accumulation of DC, *o*-Q and D in the reaction medium at short times. B. Curves of O_2 and M consumption rates (V^{M,O_2}) and ($V^{M,M}$) and DC formation ($V^{M,DC}$) at short times. C. Evolution of the concentration of the different enzyme species over time: Eox, Em and EmM. D. Variation of D concentration with time. The experimental conditions are the same than in Figure 2.

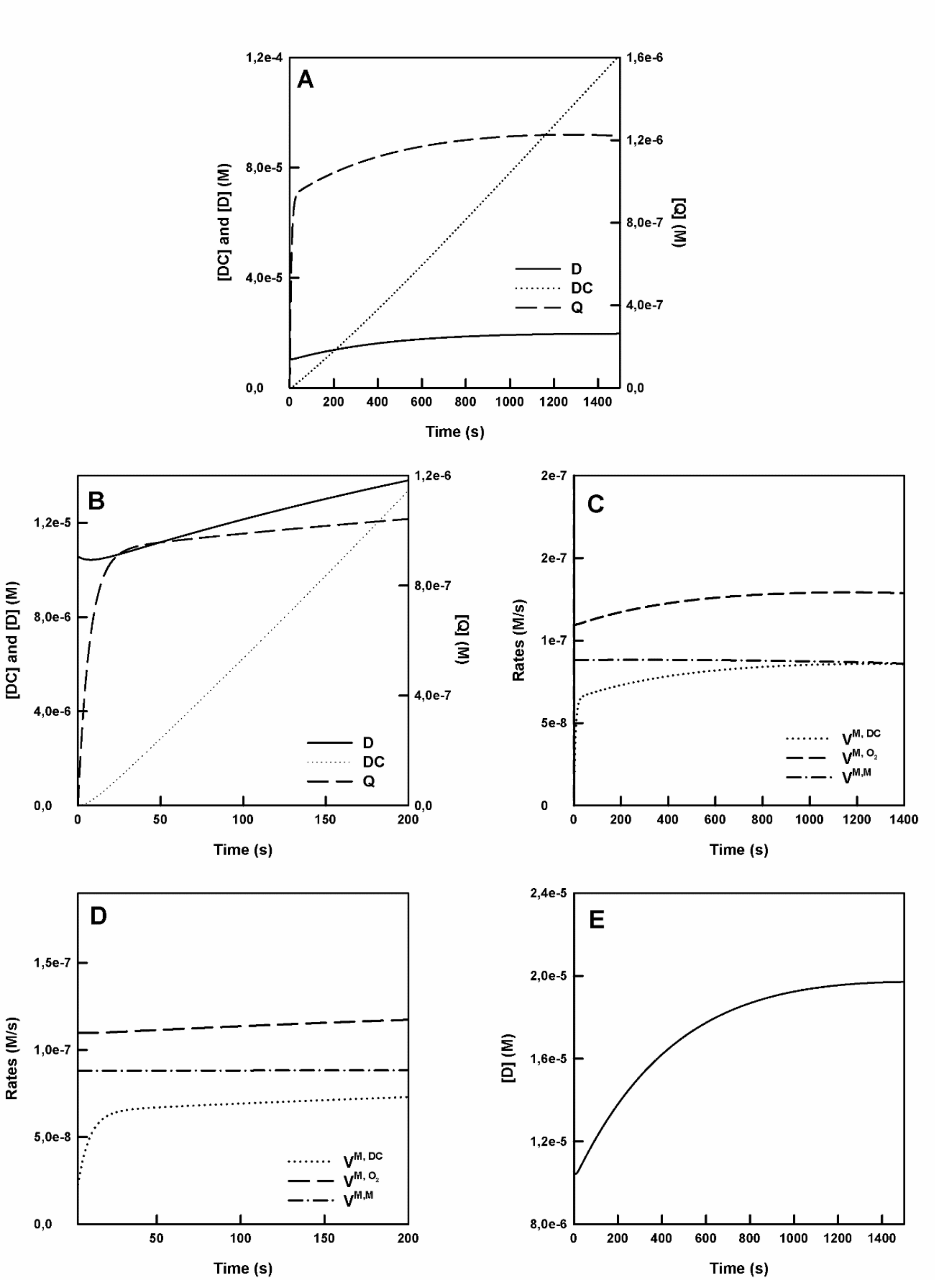

Figure S3. Simulated curves of the action of tyrosinase on L-tyrosine, a concentration of *o*-diphenol is added to the medium lower than that necessary to reach steady-state. A. Accumulation curves of DC, *o*-Q and D in the medium. B. Accumulation curves of DC, *o*-Q and D in the medium to short time. C. Relationship between the rates of consumption of O_2 (V^{M,O_2}), M ($V^{M,M}$) and of formation of

DC ($\mathrm{V}^{\mathrm{M,DC}}$). D. Relationship between the rate of consumption of M ($\mathrm{V}^{\mathrm{M,M}}$) and of formation of DC ($\mathrm{V}^{\mathrm{M,DC}}$) at short reaction times. E. Variation of the D concentration with time. The experimental conditions are the same than in Figure 1, but in this case, $[\mathrm{D}]_0 = 1.057\mathrm{x}10^{-5}\ \mathrm{M}$.

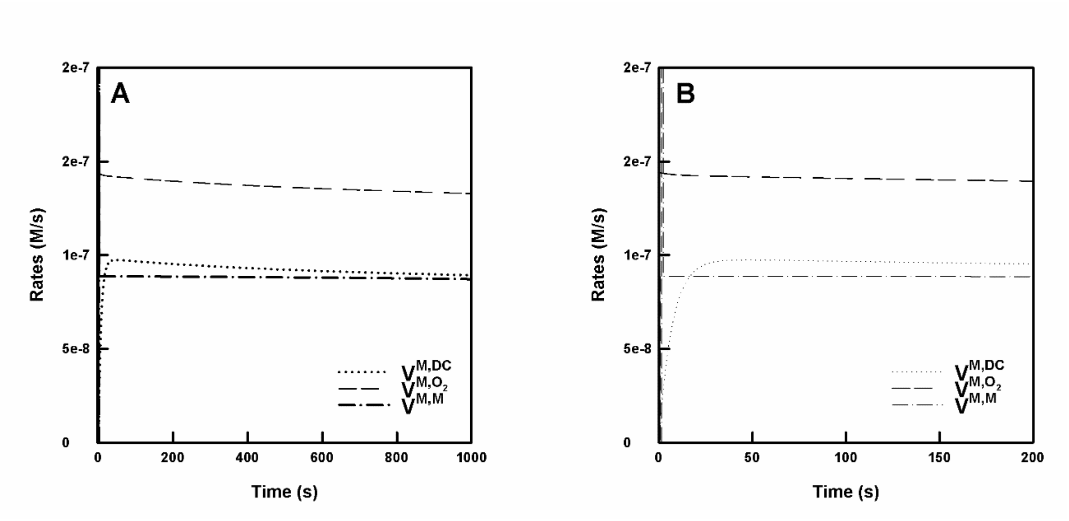

Figure S4. Simulated curves of the action of tyrosinase on L-tyrosine, a concentration of *o*-diphenol is added to the medium greater than that necessary to reach the steady-state. A. Relationship between the consumption rates of: O_2 (V^{M,O_2}), M ($V^{M,M}$) and that of DC formation ($V^{M,DC}$). B. Relationship between rates at short times. The experimental conditions are the same than in Figure 1, but in this case, $[D]_0 = 2.642x10^{-5}$ M.

Table S1. Kinetic characteristics of the action of tyrosinase on different monophenols and spectroscopic properties of the oxidized adducts formed with MBTH.

Substrate	Assay method	λ_{max} (nm)	λ_i (nm)	ε_{max} x10^{-3} ($M^{-1}cm^{-1}$)	ε_i x10^{-3} ($M^{-1}cm^{-1}$)	K_m^M (mM)	k_{cat}^M (s^{-1})	δ_4 (p.p.m)	Reference
4HA	MBTH	492	464	31.3 ± 0.8	19.2 ± 0.4	0.08 ± 0.003	184 ± 6	152.29	[11–13]
PHPPA	MBTH	500	466	40 ± 1	20.0 ± 0.5	0.44 ± 0.01	67 ± 3	156.13	[5]
Tyramine	MBTH	503	476	42 ± 1	20.7 ± 0.4	0.51 ± 0.02	26 ± 1	157.28	[14]
Tyrosine	MBTH	507	484	38 ± 1	22.3 ± 0.5	0.21 ± 0.01	7.9 ± 0.3	158.86	[11]

4HA = 4-hidroxyanisol; PHPPA = p-hydroxyphenyl propionic acid; λ_i = wavelength of the isosbestic point generated in the evolution of the adduct; K_m^M = Michaelis constant for the monophenol; k_{cat}^M = catalytic constant of tyrosinase action on monophenol. δ_4 = chemical displacement in ^{13}C for the carbon C-4.

References

1. Muñoz-Muñoz, J.L.; Garcia-Molina, F.; Varon, R.; Garcia-Ruíz, P.A.; Tudela, J.; Garcia-Cánovas, F.; Rodríguez-López, J.N. Suicide inactivation of the diphenolase and monophenolase activities of tyrosinase. *IUBMB Life* **2010**, *62*, 539–547, doi:https://doi.org/10.1002/iub.348.
2. Rodríguez-López, J.N.; Ros, J.R.; Varón, R.; García-Cánovas, F. Oxygen Michaelis constants for tyrosinase. *Biochem. J.* **1993**, *293*, 859–866, doi:10.1042/bj2930859.
3. Varon, R.; Garcia-Sevilla, F.; Garcia-Moreno, M.; Garcia-Canovas, F.; Peyro, R.; Duggleby, R.G. Computer program for the equations describing the steady state of enzyme reactions. *Bioinformatics* **1997**, *13*, 159–167, doi:10.1093/bioinformatics/13.2.159.
4. Rodríguez-López, J.N.; Fenoll, L.G.; García-Ruiz, P.A.; Varón, R.; Tudela, J.; Thorneley, R.N.F.; García-Cánovas, F. Stopped-Flow and Steady-State Study of the Diphenolase Activity of Mushroom Tyrosinase. *Biochemistry* **2000**, *39*, 10497–10506, doi:10.1021/bi000539+.
5. Espín, J.C.; Morales, M.; García-Ruiz, P.A.; Tudela, J.; García-Cánovas, F. Improvement of a Continuous Spectrophotometric Method for Determining the Monophenolase and Diphenolase Activities of Mushroom Polyphenol Oxidase. *J. Agric. Food Chem.* **1997**, *45*, 1084–1090, doi:10.1021/jf960428a.
6. Espín, J.C.; Morales, M.; Varón, R.; Tudela, J.; García-Cánovas, F. Continuous Spectrophotometric Method for Determining Monophenolase and Diphenolase Activities of Pear Polyphenoloxidase. *J. Food Sci.* **1996**,

61, 1177–1182, doi:https://doi.org/10.1111/j.1365-2621.1996.tb10955.x.

7. García-Molina, F.; Muñoz, J.L.; Varón, R.; Rodríguez-López, J.N.; García-Cánovas, F.; Tudela, J. A Review on Spectrophotometric Methods for Measuring the Monophenolase and Diphenolase Activities of Tyrosinase. *J. Agric. Food Chem.* **2007**, *55*, 9739–9749, doi:10.1021/jf0712301.
8. Garcia-Carmona, F.; Garcia-Cánovas, F.; Iborra, J.L.; Lozano, J.A. Kinetic study of the pathway of melanizationn between l-dopa and dopachrome. *Biochim. Biophys. Acta - Gen. Subj.* **1982**, *717*, 124–131, doi:https://doi.org/10.1016/0304-4165(82)90389-0.
9. Winder, A.J.; Harris, H. New assays for the tyrosine hydroxylase and dopa oxidase activities of tyrosinase. *Eur. J. Biochem.* **1991**, *198*, 317–326, doi:https://doi.org/10.1111/j.1432-1033.1991.tb16018.x.
10. García-Sevilla, F.; Garrido-del Solo, C.; Duggleby, R.G.; García-Cánovas, F.; Peyró, R.; Varón, R. Use of a windows program for simulation of the progress curves of reactants and intermediates involved in enzyme-catalyzed reactions. *Biosystems* **2000**, *54*, 151–164, doi:https://doi.org/10.1016/S0303-2647(99)00071-4.
11. Espín, J.C.; Varón, R.; Fenoll, L.G.; Gilabert, M.A.; García-Ruíz, P.A.; Tudela, J.; García-Cánovas, F. Kinetic characterization of the substrate specificity and mechanism of mushroom tyrosinase. *Eur. J. Biochem.* **2000**, *267*, 1270–1279, doi:https://doi.org/10.1046/j.1432-1327.2000.01013.x.
12. Espín, J.C.; Varón, R.; Tudela, J.; García-Cánovas, F. Kinetic study of the oxidation of 4-hydroxyanisole catalyzed by tyrosinase. *IUBMB Life* **1997**, *41*, 1265–1276, doi:https://doi.org/10.1080/15216549700202361.

13. Espín, J.C.; Tudela, J.; García-Cánovas, F. 4-Hydroxyanisole: The Most Suitable Monophenolic Substrate for Determining Spectrophotometrically the Monophenolase Activity of Polyphenol Oxidase from Fruits and Vegetables. *Anal. Biochem.* **1998**, *259*, 118–126, doi:https://doi.org/10.1006/abio.1998.2598.
14. Rodriguez-lopez, J.N.; Escribano, J.; Garcia-canovas, F. A Continuous Spectrophotometric Method for the Determination of Monophenolase Activity of Tyrosinase Using 3-Methyl-2-benzothiazolinone Hydrazone. *Anal. Biochem.* **1994**, *216*, 205–212, doi:https://doi.org/10.1006/abio.1994.1026.
